# A Community-Engaged Approach to Understanding Suicide in a Small Rural County in Georgia: A Two-Phase Content Analysis of Individual and Focus Group Interviews

**DOI:** 10.3390/ijerph20247145

**Published:** 2023-12-05

**Authors:** Kimberly Beth Roth, Eleni Gaveras, Fatima Ghiathi, Eric Kendall Shaw, Melanie Shanlin Shoemaker, Nicholas Adam Howard, Meena Dhir, Genesis Rebeca Caiza, Hannah Selene Szlyk

**Affiliations:** 1 Department of Community Medicine, School of Medicine, Mercer University, 1250 E 66th Street, Savannah, GA 31404, USA; fatima.a.ghiathi@live.mercer.edu (F.G.); shaw_ek@mercer.edu (E.K.S.); melanie.shanlin.shoemaker@live.mercer.edu (M.S.S.); ahoward2346@gmail.com (N.A.H.); meena.dhir@live.mercer.edu (M.D.); genesis.rebeca.caiza@live.mercer.edu (G.R.C.); 2 Brown School of Social Work, Washington University in St. Louis, 1 Brookings Drive, St. Louis, MO 63130, USA; eleni@wustl.edu; 3 Department of Psychiatry, School of Medicine, Washington University in St. Louis, 660 South Euclid Avenue, St. Louis, MO 63110, USA; szlyk@wustl.edu

**Keywords:** community-engaged, suicide prevention, rural, qualitative, mental health

## Abstract

Suicide is a significant public health problem, with disproportionate rates in rural areas. Rural communities face substantial structural and cultural barriers to suicide prevention. This study aimed to gain a deeper understanding of the need for suicide prevention and gauge the appropriateness of prevention efforts in the context of a rural Georgia county by leveraging existing community resources and knowledge. Twenty one-on-one, semi-structured interviews and two focus groups were conducted, with participants recruited via purposive snowball sampling. Data analysis included qualitative deductive and inductive content analysis from individual interviews and focus groups with community stakeholders. The findings highlight how rural contexts exacerbate drivers of death by suicide and how the substantial loss of community members to suicide contributes to the ongoing crisis and reduces available support. Access to mental health care often depended on a connection to an established public system such as schools, a military base, or Veterans Administration. There were perceived gaps in crisis and post-crisis services, with participants actively trying to address these gaps and build community support through coalition building. This study contributes knowledge to contextual drivers of suicide in rural areas beyond individual-level risk factors. Community-engaged suicide prevention research in rural areas is promising, but there is a need to develop interventions to best support coalition building and capacity development.

## 1. Introduction

Despite public health efforts, US suicide rates have been increasing steadily over the last 20 years, with steeper increases occurring more recently [1,2]. Data from the 2020 National Survey on Drug Use and Health indicated that among adults aged 18 or older in 2020, 12.2 million people had serious thoughts of suicide, 3.2 million people made a suicide plan, and 1.2 million people attempted suicide in the past year. In 2020, of those between the ages of 12 to 17, 3.0 million people had serious thoughts of suicide, 1.3 million people made a suicide plan, and 629,000 people attempted suicide in the past year [3]. Suicide is the second leading cause of death among ages 10 to 34, the fourth leading cause among ages 35 to 54, and the eighth for adults aged 55 to 64 [4]. This trend has been observed for all age groups, although in recent years the rates for young adults (ages 25–44) have surpassed that of adults aged 65 and older [1,5]. 

Rural communities in the US bear a disproportionate burden when it comes to suicide mortality, as rates are higher in rural areas than in urban ones [6]. Further, rates have been increasing more quickly in rural areas [7,8], contributing to widening rural health disparities [6]. The difference in suicide rates between rural and urban areas has widened from 1999 to 2017, increasing by 53% in rural areas when compared to only 16% in urban areas [9]. Specifically, suicide rates in rural areas have been higher and have continued to accelerate more quickly since 2007 [10]. Georgia is no exception to this trend, with 2021 data revealing an age-adjusted death rate due to suicide of 18.4 per 100,000 persons in rural Georgia counties compared to 14.2 in non-rural counties [11]. 

Suicide mortality reflects only a fraction of the burden on individuals and communities. Suicidal ideation and plans often lead to attempts, which are costly and more numerous than suicide deaths [1,12]. In 2018, almost 450,000 adults were hospitalized on at least one night for a suicide attempt—almost 10 times the number of deaths [12]. Additionally, 717,000 adults received medical attention for an attempt, and almost 1.5 million adults self-reported an attempt [12]. Therefore, when assessing the burden of suicide in a community, it is essential to consider suicidal ideation and behavior, in addition to suicide mortality. Unfortunately, regular and reliable surveillance data on non-lethal suicidal behavior are usually not available for rural areas, but it is probable that this disparity exists for suicidal ideation and behavior as well.

In addition to higher suicide rates, rural areas face unique contributors to suicide and barriers to suicide prevention in the form of cultural, social, and economic factors [10]. The aforementioned increased rates in rural counties have been linked to higher deprivation, higher social fragmentation, lower social capital, the higher availability of gun shops, and a greater proportion of veterans and an uninsured population residing within a county [7]. Culturally, access to firearms likely plays a role in elevated rural suicide rates. One study on gun owners found that those who owned a firearm for protection were less likely to engage in lethal means safety practice, more likely to store their firearm loaded, and were less likely to believe that firearm ownership and storage practices were linked to the risk of suicide [13], highlighting the importance of gun culture in rural areas. 

Structural barriers to accessing care are also important. For example, access to specialty mental health care providers is limited due to the sparse number of providers in rural areas, as well as the lack of transportation [14,15]. This can affect suicide, as one US study found that for every 10% increase in the mental health workforce, there was a 1.2% reduction in firearm suicides [16]. Access to emergency medical facilities, often the place where suicide attempts are treated, is also limited [17].

Incorporating qualitative research into suicide prevention is essential, as it can illuminate the complex constellation of risk and protective factors that affect suicidal acts and behavior [18]. Qualitative research can also offer deeper understandings of the structural contexts (e.g., geographical, policy, service use landscape) in which prevention efforts take place [18]. This stands in contrast to quantitative studies, which often focus on explanation of phenomena (e.g., causal relationships) [18]. Unfortunately, qualitative studies reporting community-based suicide prevention efforts are sparse, particularly in the US. Existing international work underscores barriers, such as stigma or aspects of rural culture [19,20], or reinforces addressing barriers, such as healthcare shortages [21]. Efforts must be community-led and engage the entire community, not just clinical professionals, leveraging partnerships [22]. Such work must identify and address self-identified community needs [22], but also use trauma-informed strategies [19]. 

### Role of Community-Based Suicide Research

Suicide is a multi-faceted and multi-level problem; therefore, a social–ecological perspective [23] is useful when trying to understand its determinants in a specific community context. The Suicide Prevention Resource Center (SPRC) recommends a comprehensive approach to suicide prevention for it to be effective, which typically requires “a combination of efforts that work together to address different aspects of the problem” [24]. It contains several strategies, programs, and practices to consider, spanning several socio-ecological levels (i.e., from individual to systems-level solutions) [23]. 

Tailoring preventive interventions to specific contexts and communities is essential, yet often this crucial step is overlooked [25]. Although the available county-level data can provide some broad insight into possible contributing factors (e.g., high poverty and unemployment rates), qualitative data from a community perspective can provide a deeper and more nuanced understanding of who in the community is struggling and potential ways to support them. Qualitative data provide a rich picture of the community context from the perspective of community members, so that any suicide prevention effort will be appropriate for that community. 

This study gathered the necessary formative research through in-depth qualitative interviews and focus groups to successfully adapt the most appropriate preventive intervention to the population and context. Specifically, we aimed to: (1) gain a deeper understanding of the perceived problem as it relates to suicide and suicidality in the county; (2) understand the unique community context of the county as it relates to suicide and suicidal behavior, with a focus on identifying previous or existing suicide prevention efforts in county and key informants who are instrumental in implementing or enhancing such efforts; and (3) assess the feasibility and accessibility of implementing different rural suicide prevention efforts, including identifying barriers to their success. This formative research project will lay the essential groundwork for future prevention work in which a feasible and acceptable suicide preventive intervention will be implemented to reduce suicide and suicidal behavior in the identified high-risk group(s). 

## 2. Materials and Methods

**Figure 1:**
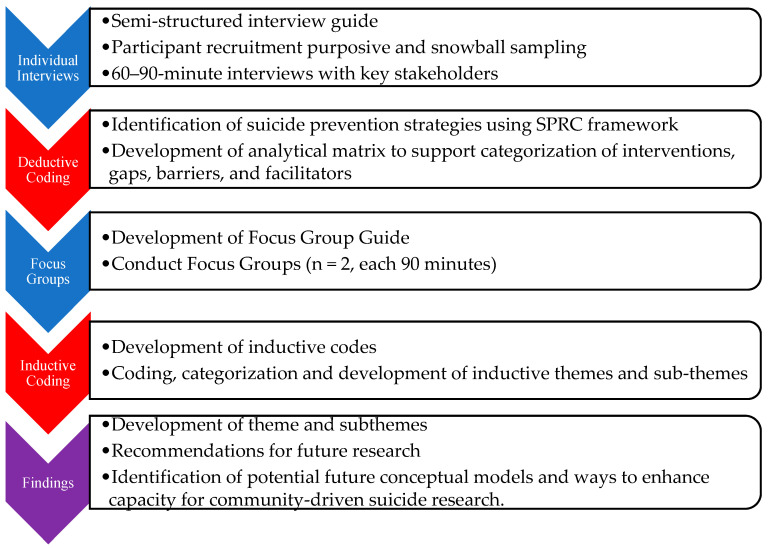
Flow Chart of Study Data Collection and Analysis Processes. Note: data collection activities are shown in blue and analyses are shown in red.

This is a community-based qualitative study using both in-depth interviews and focus groups. Figure 1 displays a visual flow chart of the data collection and analysis process. 

### 2.1. Setting

The study county, located in Georgia, is a relatively small, rural county with a military base that is a prominent part of the local economy and county members’ livelihood. This county is no exception to the disproportionately high suicide rates in rural areas, ranking fourteenth in suicide mortality in the state [26]. Suicide was in the top 10 leading causes of death in the county in 2015–2019, and ranked as the top cause of years of potential life loss [26], indicating that younger residents are dying by suicide. In 2019, there were 15 suicide deaths, amounting to a death rate of 24.4 per 100,000 residents—60% higher than the state average [26]. The suicide rate has increased 2.5-fold since 2000, an increase much more dramatic than that observed for the state as a whole [26]. Because of the lack of additional county-wide data, it is essential to gain a deeper understanding of the scope of the problem (regarding fatal and non-fatal suicidal behavior) in the community and the subgroups within the community that are experiencing the highest need in order to identify the most appropriate program or intervention to address the high suicide rates in the county.

### 2.2. Participants and Data Collection

Data collection occurred from August 2021 to June 2022.

Individual data collection consisted of 20 semi-structured one-on-one interviews with 22 community stakeholders, purposively selected from the community and through snowball sampling. Recruitment involved contacting potential participants by email and/or phone. In order to be eligible for participation, individuals had to be over 18 years of age, living and/or working in the study county, and interested in discussing the topic of suicide in the community. To ensure coverage across the entire community, we identified five broad sectors of stakeholders and contacted individuals with leadership positions in those sectors who might have insight into the problem. We asked all participants, regardless of willingness or ability to participate, to recommend additional community members that we should talk to. Interview participants primarily came from the non-profit/religious sector (n = 7, 32%), followed by the healthcare (n = 6, 27%), education (n = 5, 23%), military (n = 2, 9%), and government (n = 2, 9%) sectors. The majority of participants were female (73%), with an average age of 51.7 years of age. Fifteen individuals were living in the county (an average of 19.1 years of residence), and six worked in the county but lived elsewhere (an average of 12.5 years worked). 

Senior members of the research team (who have expertise in suicide prevention, qualitative research, psychiatric epidemiology, social work, sociology, and lived experience in suicide prevention research) developed a semi-structured interview guide to align with our goal of gaining a deeper understanding of the scope of the problem, including which members of the community are struggling and the community context. We explored interviewees’ views on contributing and protective factors that may affect the high suicide rate and what they want to see changed in their community. Interviews were carried out by two members of the senior research team and research assistants. Research assistants were medical students at Mercer University from a wide range of backgrounds, including both rural and urban contexts, including rural Georgia. Prospective participants were not contacted more than three times. Interviews were conducted either in person or via Zoom, depending on the interviewee’s preference, and lasted approximately 60 to 90 min. All interviews were recorded and later transcribed by a member of the research team using Transcribe [27]. Interviewers memoed their experience post interview to allow the research team to ground the context in which the data were collected. 

Focus group members were recruited from participants in the individual interviews as well as other stakeholders interested in implementing a community-based suicide prevention effort, as identified through the in-depth interviews. All interviewees were invited to participate in the focus groups. Snowball sampling was also employed for focus group recruitment. Focus group guides were informed through the deductive analysis of individual interviews and reviewed by senior members of the research team. Focus groups consisted of 6 to 10 participants who identified as a stakeholder in suicide prevention efforts in the community. Sessions lasted approximately 1.5 h, were conducted in person, and were led by two members of the research team. Sessions were audio recorded and transcribed in the same manner as the semi-structured interviews. In addition to audio capturing the focus group sessions, a third research team member memoed their observations, interactions, and experiences during the focus group sessions. Participants received a USD 50 Amazon gift card for their participation in the interviews or focus groups. 

#### Ethical Considerations

All research activities in the present study adhered to the principles set forth in the Declaration of Helsinki, which guide research involving human subjects. Informed consent was obtained for participation in interviews and focus groups, and participation was voluntary. All procedures were approved by the Mercer University Institutional Review Board (#H2107137_01).

### 2.3. Data Analysis 

Data were analyzed using a two-step deductive and inductive content analysis adapted from Elo and Kyngäs [28] and Sandström et al. [29] (see Figure 1). Content analysis is a flexible analysis method that involves the distilling of textual data into categories or themes and is helpful for drawing connections from data to context, simplifying a broad phenomenon, and developing new insights and actions [28]. This process allowed development of themes and subthemes that are both conceptually driven (deductive) and derived from the data (inductive) [30,31]. Both rounds of coding were conducted in Dedoose 9.0.17 [32]. 

**Figure 2:**
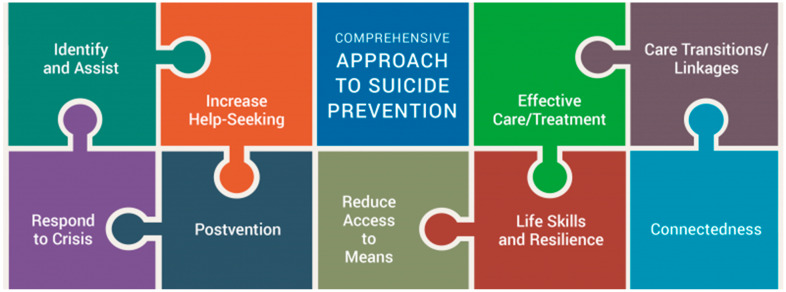
Elements of a Comprehensive Approach to Suicide Prevention. Note: taken from the Suicide Prevention Research Center’s website: https://sprc.org/effective-prevention/comprehensive-approach (accessed on 21 September 2023).

Phase I deductive coding used the SPRC’s Comprehensive Approach to Suicide Prevention (hereafter, SPRC framework) to distill the data into more concise meaning units or excerpts [33] and organize these excerpts into one of nine intervention types identified by the SPRC framework (see Figure 2 for the nine intervention types). Members of the study team then conducted a sorting exercise adapted from Sandström et al. [29], in which excerpts were sorted into whether they described existing resources/facilitators or barriers/gaps or could not be sorted. Reflections on the exercise and excerpts that could not be sorted and why were then discussed with members of the research team. Deductive findings contributed to materials for the focus group discussion, where participants were presented with intervention types that came up most often and sample quotes for different intervention types. 

Phase II inductive codes were developed through memoing and discussion during the deductive Phase I of coding. The process of memoing in qualitative data collection and analysis allows the researchers to reflect on the process, helps shape codes and themes, and allows for further thought about new connections to facilitate deeper understanding [34]. Additionally, open coding of the focus groups contributed to the refinement of the codes and sorting codes into categories, subthemes, and themes. The development of findings was an iterative process in which researchers discussed each step of the data collection and analysis and how they built upon each other, as well as what is known from the literature. As part of the entire iterative process, the authors reflected on their own positionality in relation to the data as mental health and community health researchers and medical students, as well as how their own biases toward desired outcomes and perspectives may have affected the interpretation of the data. 

## 3. Results

Three themes emerged from the data: (1) cumulative trauma and isolation; (2) support networks and systems; and (3) treatment gaps and community response (Table 1). **Theme 1, cumulative trauma and isolation,** illuminates perceived contextual drivers of suicide in the county, including how individuals, families, and the county are responding to crisis and geographic isolation (subtheme 1.1), as well as a substantial loss of life due to death by suicide in the community. **Theme 2, support networks and systems,** illustrates how access to suicide prevention in rural areas is not universal but dependent on connection to formal (i.e., health and mental health care; subtheme 2.1) and informal (i.e., family and friends) systems and resources (subtheme 2.2). Finally, **Theme 3, treatment gaps and community response,** illustrates gaps in long-term mental health care, crisis services, and postvention (subtheme 3.1) and grassroots community efforts to provide community and postvention support (subtheme 3.2).

### 3.1. Theme 1: Cumulative Trauma and Isolation

In this theme, participants gave insight into the perceived contextual drivers of suicide. Two subthemes, 1.1 community crisis and 1.2 survivors of suicide loss, illustrate how the rural county context exacerbates known drivers of suicide, such as exposure to family violence, trauma, substance use, and economic crisis. Specific rural contexts that are explored in this theme include isolation, rural-specific patterns in substance use and economic crises (e.g., homelessness), and small social networks with high proportions of suicide loss survivors. In addition to exposure to known risk factors, these contexts also contributed to barriers of known protective factors, such as connectedness and social support, reduced capacity for resilience, as well as a need for increased community postvention services. Postvention involves activities, such as **providing evidence-based services**, to reduce risk and facilitate healing **in the wake of a suicide death**.

#### 3.1.1. Subtheme 1.1: Community Crisis

Exposure to violence, trauma, and economic crises were discussed as drivers of suicide risk in this county, which is reflective of what is currently known about suicide risk factors. For example, a mental health provider described a context of an unresolved crisis in the following quote:

*“Matter of fact, we’ve coined a phrase here in our practice…It’s just something we’ve used specifically, not just for [county name] County but the surrounding area, and we’ve called it “CUTI”. And we just—it’s one of those code words. We know what we’re talking about in-house. But it’s “Cumulative Unresolved Trauma Isolation.””*—ID 113. Mental health provider.

Other interviewees expanded on this idea of CUTI and illustrated perceived drivers of suicide, multigenerational exposure to childhood and domestic violence, and substance abuse exacerbated by the opioid epidemic.

Participants perceived older adolescents and young adults as the most at risk, and several stakeholders mentioned there may be a shift to younger adolescents and children. In addition to a family crisis and childhood trauma, family poverty and economic vulnerabilities were seen to exacerbate this risk. As a first responder describes:

*“It’s the ones that are living in a trailer, living in a pop-up camper with a tarp over the roof cuz there’s holes. And then they got to push it up. It’s causing a lot of depression. And then the money comes in that’s supposed to go for the kids and it’s going to alcohol. So, parents are shoving their kids out at 18. And, I mean, we had a 19-year-old that shot himself in the face because he wasn’t ready for the world.”*—ID 105. First responder.

Many other participants also cited poverty as a driver for suicide risk in the county. Risks associated with poverty included housing insecurity, inability to pay for mental health services or transportation to services, and hopelessness due to unemployment. 

Geographic isolation is also perceived to contribute to a lack of crisis services. In a discussion on crisis response, a fire department member discussed challenges when providing crisis service as a first responder. These challenges include negotiating with individuals and family members about fears of going to the hospital, the need to go to the emergency room, and other interpersonal conflicts. While there are crisis services available in Georgia, according to the fire department member, geographic isolation limits the availability of a crisis team:

*“… we have the Georgia crisis line, but in many cases, they have three or four hours of travel time to send a crisis team. So, it really puts the people that are the boots on the ground, dealing with these individuals on a daily basis, in a quandary.”*—ID 110. Fire department member.

Further, participants discussed how there are no inpatient facilities within the county, which increases transportation time and limits the ability of supportive family and community members to visit. This participant mentions that navigating this crisis puts first responders in a difficult position when assisting people navigating these crisis and emergency resources, on top of being exposed to trauma during the course of their work. As will be discussed in the next subtheme, suicide loss contributes to accumulative loss, which does not exclude service providers. 

#### 3.1.2. Subtheme 1.2: Survivors of Suicide Loss 

The loss of clients, family, and community members to suicide experienced by participants was pervasive in the data. For example, one participant noted:

*“I’ve dealt myself in the last two years with 21 area suicides. Families after the fact. Of those 21, half were in [this] county.”*—ID 113. Mental health provider.

Thus, exposure to trauma and suicide death can contribute to ongoing community crises. 

Knowing someone who has died by suicide increases the risk of death by suicide, particularly if it is a partner or other family member [35]. Participants perceived that **small communities and close-knit social networks contributed to a higher proportion of survivors of suicide loss in this region across families, peers, healthcare providers, and first responders**.

*“70,000 people in this town. Everybody’s related to somebody, and everybody’s related to somebody who’s killed themselves, and it matters…When you’re looking at numbers and statistics, it doesn’t matter. When you’re sitting there and you’re looking at the list, and you’re reading down the list and realize, “Oh my gosh. There’s my cousin’s daughter.””*—ID 120. Grassroots organization founder.

Participants also described how suicide loss impacted family support resources and provider burnout. 

### 3.2. Theme 2: Support Networks and Systems 

Theme 2 illustrates how a connection to formal public systems (subtheme 2.1) and reduced availability of family resources, including knowledge and finances (subtheme 2.2), contribute to access to suicide prevention interventions. This theme illustrates perceived supportive resources in the county, with many evidence-based interventions mentioned by providers in schools or connected with the military base/VA combined. Many providers mentioned gaps in family resources financially and socially, with stigma as a salient barrier to accessing care. Thus, this theme expands upon the contextual drivers of death by suicide (Theme 1) and illustrates how it is not only exposure to trauma that reduces resources; there are also larger implications for suicide prevention access. Deductive coding highlighted specific SPRC framework areas salient to this theme, largely implemented in schools: identify and assist, crisis response, long-term mental health care, life skills and resilience, and connectedness and social support. 

#### 3.2.1. Subtheme 2.1: Connections to Formal Systems 

**This subtheme** illustrates how, in this context, connections to the military or public-school system facilitate access to resources. A non-profit staff member describes how access to services varies by the systems community members have access to:

*“I don’t know, I think it depends on if their motivation, their support network and systems—It’s hard to give kind of a blanket response. I think that’s kind of, for each individual, it’s probably a kind of different, and dependent on a lot of factors as well. Folks who are more connected to formal organizations, such as military personnel, and because they didn’t report to duty or something they—it may become more known, have probably a greater chance of accessing help than some others or a child who confides in a peer at school, who reaches out to a counselor or teacher…”*—ID 117. Non-profit staff.

Within these systems, an individual in crisis has a much greater chance of accessing a person with training, including peers who have been trained in peer support and gatekeeper interventions.

For example, a school counselor describes ongoing efforts at the district level to provide training as a response to recent student deaths by suicide.

*“We as a district—all of our counselors just went through a suicide first aid training. …of course, all of us are trained in suicide prevention in and of itself, just from the credentials we have to have to do the job. But just from the recent deaths by suicide that we have had, there was definitely a need to say, “Okay, what else can we do?” And so, we just went through an intensive training to refresh our skills…just so that we could be more prepared to deal with our students as they are dealing with their issues…”*—ID 106. School counselor.

Crisis response and inpatient availability also varied by whether or not someone was a service member or civilian.

As stated by another school counselor: *““And that may be because a lot of what I’m dealing with is military, and they’ve got the services. And they’ve got the insurance. And they’ve got an avenue. I would say that’s probably the biggest difference.” According to key stakeholders, some of these services included increased awareness campaigns, emergency department and inpatient beds on military bases, and family counseling.”*—ID 102. School counselor.

The number of suicide prevention programs implemented in schools and the military base/VA included various types of interventions. Study participants mentioned suicide awareness programs, trained or certified staff in suicide prevention screening, long-term mental health treatment such as counselors in schools, and manualized programs to identify social support and increase coping skills. Evidence-based manualized interventions mentioned by providers included Sources of Strength [36] (school-based, addressing increasing connections and social support), ASIST (key stakeholder training) [37], Question, Persuade, Refer (QPR; gatekeeper training) [38], and units on social–emotional learning (e.g., life skills and resilience). Participants also mentioned awareness-raising campaigns in schools, such as orange shirt days and information sessions after losing a student to suicide. There were some community awareness-raising events described, such as the American Foundation for Suicide Prevention’s Out of the Darkness Walks [39] and a walk for suicide prevention among first responders. Even so, participants expressed a need to expand stigma reduction and awareness interventions to the wider community. 

#### 3.2.2. Subtheme 2.2: Barriers to Help-Seeking: Family Resources and Stigma

**This subtheme** illustrates how family resources can impact access to suicide prevention programs. Barriers to treatment included knowledge barriers and financial barriers. Knowledge barriers manifested as the lack of knowledge of existing mental health resources. Financial barriers included paying for services, as well as needing to have available transportation. Participants discussed how stigma influences access to important interventions, including connections and social support, help-seeking, and crisis services. 

Stigma was also perceived to create barriers to help-seeking and supportive connections. Stigma is defined as the societal devaluation of an identity that is perceived to be less capable, trustworthy, and ultimately less deserving than others [40]. Participants discussed how stigma influences access to important interventions, including connections and social support, help-seeking, and crisis services. Additionally, several participants mentioned cultural barriers to seeking help from family, such as being seen as weak or not wanting to talk about something uncomfortable. For example, a counselor discusses their perception of the influence of southern culture on stigma:

*“Now, my parents wouldn’t—but I know there’s people that I grew up around them that are their age [60s], that would probably not know what to do if they had an adult child tells them that they were having a problem. It’s just something that they wouldn’t talk about. It’s almost kind of like, not the southern, that kind of southern way of like not talking about things that make people uncomfortable.”*—ID118. Counselor.

While key stakeholders did mention several awareness-raising campaigns and events, they noted that the effect of these interventions often wore off over time or did not reach enough members of the community. 

Stigma combined with financial resources was often mentioned in combination as a barrier to long-term mental health resources. As described by a mental health counselor:

*“So, I think access to mental health is a big deal whether they know if it’s okay, the stigma piece, or whether they can afford it and whether they just can’t get here and looking for alternatives to try and help them to access the help that is here. So, you asked me three reasons, I would say access to mental health treatment, economic life you know just financial concerns.”*—ID112. Counselor.

As discussed more in the next section, stigma and resource constraints impacted the options that an individual having thoughts of suicide could go to. A government official discussed a combination of cost, culture, and lack of places to go to just talk to someone and receive support. Responding to a question about barriers to services:

*“Yeah, I think one is cost. I think culture, because you’re viewed as weak—in the black community especially. And I think in some of the other communities if you are talking about harming yourself. And I think another barrier is there’s no facilities! There’s no real place to go. I mean, there’s a place for drug and alcohol abuse, sexual abuse. There’s no real place for people who are feeling like they want to end it all, to go and have a group meeting, and talk about it. Not to my knowledge.”*—ID101. Government official.

This element of having no place to go to just talk without stigma or cost is further explored in Theme 3 as an area for future interventions. 

### 3.3. Theme 3: Treatment Gaps and Community Response

Theme 3 highlights gaps in suicide prevention interventions (subtheme 3.1) in the county and how community stakeholders aim to fill these gaps (subtheme 3.2). As discussed in Theme 2, access and barriers to suicide prevention interventions described by participants were dependent on institutional and family connections. In contrast, Theme 3 illustrates gaps and needs following the development of a safety plan (i.e., a written list of coping strategies and sources of support to be used in a suicidal crisis) as part of an intervention such as ASIST [37]. The SPRC framework intervention areas of care transitions and linkages, long-term mental health care, post intervention, and responding to individuals in crisis all relate to this theme. Community-conceived responses to these gaps include strengthening community collaboration. 

#### 3.3.1. Subtheme 3.1: Linkage Gaps in Mental Health Care

**This subtheme** represents significant gaps in the broader mental health system; first responders, teachers, and healthcare providers noted gaps in inpatient settings and access to longer-term mental health treatment. In conjunction with the macro- and meso-level drivers and barriers illustrated in the previous two themes, this subtheme illuminates siloed communications between the different areas of suicide prevention interventions.

**There are multiple stakeholders in suicide prevention, and key stakeholders highlighted how different providers addressed gaps in care and linkages. For example, a primary care provider explains**:

*“Like, the patient I told you, they’d been bringing him here for—because he lives in a group home. And they can’t even get them to give him his shot… I think the doctor that used to be there a lot, he kind of left, and they have problems getting personnel to run the place, to be efficient, to be open all the time.”*—ID 119. Primary care provider

In this quote, the participant describes the challenges of providing care for a patient living with a serious and persistent mental illness by filling in gaps created by an already strained system. The provider explains that “…they’ve been bringing him here for me to give him his shot, as bad as that is…” because the specialized services aren’t accessible. These providers also mentioned working with patients who lost family members to suicide and experienced suicide loss themselves. This highlights a need to address the needs of providers when conceiving suicide prevention interventions. 

Focus groups allowed us to better understand these siloed communications between providers and how they navigate gaps in suicide prevention interventions. For example, the following exchange in the focus group captures this communication silo, as participants learn about how access to telehealth for a crisis varies by setting:

ID 123 (school counselor): *“[The ER] is our point of entry usually. A lot of times we’re going to the ER in the medical… We’re going with students or advising parents to go to the ER… And also the telehealth sometimes. We have used that before…”*

ID 110 (EMS provider): *“It flabbergasts me to hear that our school system has access to telehealth, but our emergency responders don’t. Cuz if you think about the school systems are open for this short window of the day, but we’re responding to calls 24 h a day and we don’t have that, especially for the EMS side. The fire service doesn’t, but with that EMS history and background. It blows my mind that that’s not…”*

In the focus group, the conversation continues as another mental health provider tries to understand why an EMT would be interested in having access to telehealth technology. In this context, it became apparent that first responders do not have someone to call at a local mental health treatment center in a crisis situation, but also that mental health providers were not necessarily aware of service gaps after a 911 call. Thus, this discussion highlighted the importance of multi-sector communication to develop approaches to address gaps, such as giving first responders access to telehealth crisis services instead of waiting several hours for mental health crisis services (a problem raised in Theme 1).

#### 3.3.2. Subtheme 3.2: Community Response 

The study county has experienced high rates of death by suicide. However, there was a noted gap in community postvention interventions. Thus, **Subtheme 3.2** illustrates grassroots community efforts for postvention support and survivors of suicide loss. Several participants discussed their own responses, filling the need for community-level postvention interventions. First responders are organizing peer support for first responders struggling with suicidal ideation and an awareness-raising walk. Two participants, IDs 121 and 122, founded a local grassroots organization after losing their daughter to suicide. Further, several school-based counselors discussed organizing mental health fairs after student deaths due to suicide. A school administrator discusses how her spearheading the development of a coalition emerged as her filling in a gap in postvention services:

*“And [the state suicide prevention specialist] gave me a lot of trainings or list of trainings, and then I had the other death. And then I had another one. And so, at that point, I reached out to her and I was like, “Hey, something’s really, really not right going on here.” … And at that point, she suggested that we maybe start up a coalition for our community. And with that—it’s a lot of work…. And now it’s just the part of trying to get everybody together is where I’m kind of stuck at… And so, that’s where I kind of am at this point.”*—ID 104. School administrator.

The development of a coalition presents the opportunity to have a coordinated response and reduced siloed communication. Further, there are opportunities for peer support for providers. However, it is not without challenges; 104 continues on to express concern that the schools will take on the undue burden of sustaining the coalition, combined with other challenges of organizing stakeholders. 

Focus group participants also discussed the need for a space where people can come and receive resources and support. As stated by a pastor (and grassroots community organizer), “*I don’t want to herd the cats. I want to make sure the cats have a place to go.*” The discussion of a need for a cross-sector space/coalition building area continued between the pastor and a mental health counselor:

ID 121 (pastor): *“…we need to know what the medical resources are, [healthcare director], because most of the people that are counseling. They don’t know what to do. I mean, I get it all the time. They call me all the time that they call me, “Pastor. Do I send them to [mental health facility 1] or do I send them to [mental health facility 2]? They’re both full. What do I do?” And I’m like, well—”*

ID 116 (counselor): *“There needs to be somebody that binds that bit.”*

ID 121 (pastor): *“Yeah, and we don’t know where to go, and we don’t know what to do.”*

ID 128 (healthcare director): *“And see, that’s what we’re living in too, because when we’re sending somebody, we’re usually trying to find a place where to admit them, and there’s such limited places…”*

ID 121 (pastor): *“But I think the team that we develop needs to have an understanding of how that works, because if we have a better understanding of how the healthcare side of it works, if we have that education, then we become better equipped, and we might even also be able to take some of the pressure of off the ER on a cold night.”*

The focus group sessions highlighted the core of this theme: namely, that much of the initiatives in the community were siloed and there was often a lack of communication across the crisis care continuum. However, participants were eager to address existing gaps and to create connections across sectors to further suicide prevention in the county.

## 4. Discussion 

This study used qualitative methods to conduct formative work necessary to tailor suicide prevention and postvention programming for this unique rural community. Through semi-structured interviews and focus groups, we identified three overarching themes: cumulative trauma and isolation, support networks and systems, and treatment gaps and community response. These themes highlighted important contextual drivers of suicide in the community, populations with strong supports in place, specific barriers to care, as well as a systems-level assessment of weak points in the crisis care continuum. 

Broadly, many of the identified themes and sub-themes are consistent with current literature. For example, in an era where social determinants of health are gaining more attention, our results that economic crises are perceived as drivers of community suicides underscore the importance of addressing more than individual behaviors and mental illness—two factors that have traditionally received much more attention in the literature [41,42]. This also highlights the need to consider suicide and its prevention from a socio-ecological perspective. Conceptual frameworks like Bronfenbrenner’s Ecological Systems Theory [23] provide a foundation for understanding the drivers of suicide and using a multilevel approach to address it. Our results confirm this by highlighting both drivers and supports at the family, peer, and organizational level. Finally, rural-specific factors such as stigma and lack of available providers also rose to the top in our results. These factors are barriers consistently discussed in the literature when considering mental health care in rural areas [43]. Further, the linkage gaps in the mental health care system that were discussed are representative of nationwide system gaps (e.g., staff shortage, burnout, long waitlists/lack of beds, siloed provider communication), but other factors like long travel times are more prevalent in rural communities.

Similarly, the lack of certain themes should be noted. First, there was no mention of access to firearms as a driver of suicide, or limiting access as a possible prevention strategy. This stands in stark contrast to the evidence that one of the most effective ways to reduce suicide is to reduce the lethality of attempts [10], compounded with evidence that individuals in rural areas are more likely to choose to die by firearm [44], which is the most lethal method [45]. Second, although a wide variety of factors were described when participants discussed drivers of the suicide problem in the county, mental illness was rarely explicitly mentioned as a cause of suicide. Although suicide is a multifaceted problem, many studies show that mental illness is a precursor to suicidal thoughts and behaviors [41]. Third, the lack of emphasis on postvention is striking, especially given the small, highly interconnected community. Postvention has long been promoted as a way to prevent suicide among suicide survivors—those who have lost a loved one by suicide and, as a result, are at an increased risk of suicide themselves [46]. Some have argued that postvention is so essential to suicide prevention that any program must include a postvention component [47]. 

We also saw some themes that likely arose from the unique community context of this rural Georgia county. Although we saw recurring themes that are common among rural communities (e.g., stigma, lack of healthcare providers) [43,48], the county has unique factors that affect the context in which suicide drivers and prevention take place. Most salient is the military base located within its borders, not only creating a high percentage of service members, veterans, and their families—which we know have higher suicide rates than the general population [49,50]—but also a transience and sense of isolation that respondents felt were more prominent in the county. There was also a realization of a fair number of programs available, particularly if you were connected to the school system or military, but that these initiatives and available providers were extremely siloed. 

Our findings have important implications for next steps. First, there appears to be a place for capacity building in this community. Chaskin [51] defines community capacity as “the interaction of human, organizational, and social capital existing within a given community that can be leveraged to solve collective problems and improve or maintain the well-being of a given community. It may operate through informal social processes and/or organized efforts by individuals, organizations, and the networks of association among them and between them and the broader systems of which the community is a part”. In this context, capacity is a community’s ability to address the suicide problem and/or implement and enhance suicide prevention efforts. Respondents talked about capacity in both an individual/emotional as well as an institutional/structural manner. In particular, some structural dimensions of Chaskin’s framework were highlighted, such as the current limited capacity in facilities (e.g., not enough inpatient beds) and the limited communication across sectors. In light of this, a reasonable next step would be a county-wide inventory of available services across the crisis care continuum, such as the Crisis Intercept Mapping process that the Substance Abuse and Mental Health Services Administration (SAMHSA) undertakes in for suicide prevention among service members, veterans, and their families. This is a locally driven approach to strengthen a community’s coordination of crisis care, stakeholder partnerships, and delivery of evidence-based suicide prevention policies [52]. Such an approach could help address the siloed efforts that are unevenly distributed in this county and identify gaps and barriers to crisis services.

Second, it is clear more rural-focused suicide preventive interventions and postventions are needed. The identified themes can help move these evidence-based interventions forward. Strikingly, the community must undertake this while still grieving from suicide loss. At least three different levels of responses are needed: universal prevention to inform the community about suicide risk and to help address stigma; communication pathways between providers and institutions, and resource lists for the community when intervention is needed; and community healing via postvention so that individuals can process a loss. Taken together, these things would help fill the identified gaps in a community that is actively trying to mobilize around suicide prevention.

### Limitations

Despite its strengths, this study was not without limitations. We used snowball sampling, which may lead to a biased sample, but it allowed us to recruit key stakeholders across multiple domains. A couple of interviews experienced technical difficulties, making parts difficult to transcribe due to glitches or noise. While we conducted an adequate number of interviews for a small qualitative study, we were relying on a relatively small number of people in each stakeholder domain; thus, themes arising from their subjective perspectives on the subject matter may have differed if we had interviewed more people per domain. We had a finite number of people in these different domains, and likely missed important groups. For example, military and non-mental health services were under-represented in our sample.

## 5. Conclusions

This study laid the essential groundwork to address high suicide rates in a community where stakeholders across a variety of sectors recognize the need for prevention and intervention. Knowing the unique community context is critical in order to tailor such prevention programming to meet the specific needs of a rural community, which in and of itself presents unique challenges. Although many themes support the existing knowledge base in the literature, understanding perceived drivers and existing gaps will help guide intervention selection. There is also a readiness at the grassroots level to address the problem, yet it has been difficult to gain traction in building a suicide prevention coalition. Because suicide is a multifactorial problem, it is important to take a holistic approach, incorporating multiple social–ecological levels, when building and tailoring prevention programming.

## Figures and Tables

**Table 1:** Identified themes and sub-themes.

Themes	Subthemes
1. Cumulative trauma and isolation	1.1. Community crisis 1.2. Survivors of suicide loss
2. Support networks and systems	2.1. Evidence-based practices: Connections to formal systems 2.2. Barriers: Family resources and help-seeking
3. Treatment gaps and community response	3.1. Linkage gaps in mental health care 3.2. Lack of formalized postvention services

## Data Availability

The data presented in this study are available on request from the corresponding author. The data are not publicly available to protect participant privacy.
